# Congenital Hepatic Fibrosis and Need for Liver Transplantation

**Published:** 2010-05-01

**Authors:** B. Geramizadeh, P. Keramati, A. Bahador, H. Salahi, S. Nikeghbalian, S. M. Dehghani, S. A. Malek-Hosseini

**Affiliations:** 1 *Transplant Research Center, Shiraz, Iran, *; 2 *Department of Pathology, Shiraz University of Medical Sciences, Shiraz, Iran,*; 3 *Transplant Ward and Center, Nemazee Hospital, Shiraz, Iran*

## Abstract

Herein, we describe two patients who underwent liver transplantation with the clinical diagnosis of hepatic failure and cryptogenic cirrhosis; histopathology of the explanted hepatectomy specimen revealed congenital hepatic fibrosis. To the best of our knowledge, coexistence of hepatic failure and cirrhosis in congenital hepatic fibrosis, have not yet been reported in the English literature.

## INTRODUCTION

Congenital hepatic fibrosis (CHF) refers to a unique liver histology characterized by bland portal fibrosis, proliferation of interlobular bile ducts in the portal areas with variable shapes and sizes of bile ducts [1].

Clinical progression and presentation of CHF is highly variable and symptoms may appear early or late in life [2]. Pure CHF is rare and its main consequence is portal hypertension and most commonly associated with Caroli’s disease [3].

Impaired liver function and hepatic failure leading to liver transplantation without any history of cholangitis have not yet been reported in the English literature. Herein, we report on two rare cases of CHF with different presentations of hepatic failure, who needed liver transplantation and the diagnosis of CHF was made after histopathological examination of the hepatectomy specimens.

## CASE REPORTS

Case 1:

A 33-year-old jobless man was referred to the hospital for continued management of cryptogenic liver cirrhosis. He had previously presented with ascites and esophageal variceal bleeding which was repeatedly treated with sclerotherapy. All of the investigations including tests for viral markers, autoantibodies and workup for metabolic diseases such as Wilson’s disease, iron profile and α_1_ antitrypsin deficiency were negative. There has been no family history of liver diseases; the patient had no history of alcohol intake. Also, no past history for surgery was noted. On physical examination, he had hepatosplenomegaly, mild jaundice and ascites. A complete blood count showed pancytopenia with profound thrombocytopenia; he had a hemoglobin of 11 g/dL, white cell count of 4000/µL and a platelet count of 85,000/µL. Prothrombin time was prolonged. In the first admission, liver function tests had been unremarkable but his last laboratory findings were abnormal with low albumin and high bilirubin. His last laboratory findings before liver transplantation are shown in Table 1. Renal function tests were normal.

**Table 1:** Laboratory findings in two cases of CHF at the time of liver transplantation

Lab Findings	Case 1	Case 2
Albumin	2.2 g/dL	3 g/dL
Prothrombin time	19.3 s	>30 s
AST*	44 U/L	44 U/L
ALT†	24 U/L	35 U/L
Total Bilirubin	3.1 mg/dL	4.2 mg/dL
Direct Bilirubin	1.1 mg/dL	0.5 mg/dL
BUN‡	19 mg/dL	15 mg/dL
Creatinine	1 mg/dL	1.1 mg/dL

* AST: Aspartate aminotransferase (Normal <28 U/L);

† ALT: Alanine aminotransferase (Normal<28 U/L);

‡ BUN: Blood urea nitrogen

Abdominopelvic sonography showed mildly enlarged liver with coarse parenchymal echogenicity, enlarged spleen, mild ascites, and bilateral kidney enlargement with no stone or any cystic lesions. Liver biopsy was not taken because of thrombocytopenia and coagulopathy.

Orthotopic liver transplantation (OLT) from a cadaveric liver was performed for him with the clinical diagnosis of cirrhosis and no identifiable underlying cause. His post-operative course was uneventful, except for a few episodes of mild rejection. After one year of follow-up, he is doing well.

Case 2:

A 13-year-old boy, a known case of cryptogenic cirrhosis was admitted to the hospital for OLT. He had presented with symptoms of hepatic failure and portal hypertension since five years before. He had several previous admissions of gastrointestinal bleeding secondary to esophageal varices during the last few months. All the investigations for determining the cause of cirrhosis were normal at that time. There was no family history of liver diseases.

His physical examination showed a huge splenomegaly and a hard hepatomegaly (4 cm below the costal margin). Mild jaundice was noted but there were no ascites.

Abdominopelvic sonography showed heterogenic parenchymal echogenicity of the liver with irregular border. Both kidneys were normal.

His laboratory findings are shown in Table 1. He underwent OLT with good results. After two years of follow-up, he is doing well.

The received liver specimens taken from both cases has micronodular cirrhosis in gross appearance.

**Figure 1:**
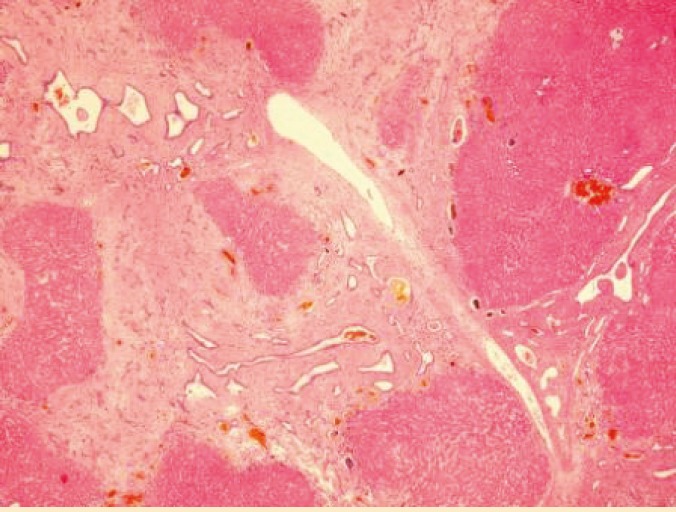
Sections from the explanted liver shows typical histology of congenital hepatic fibrosis (H&E, ×100)

Microscopic examination of the specimens showed islands of liver parenchyma which were separated by broad and narrow septa of dense and mature fibrous tissue containing elongated and irregular ductal plates. Two separate sets of ducts were present—one lying centrally in the septa and another near the parenchyma (Fig. 1).

## DISCUSSION

CHF is an uncommon malformation characterized by enlarged portal spaces containing abundant collagen and many ectatic bile ducts. The main complication is portal hypertension and bleeding from esophageal varices [3, 4].

Typically, CHF has been described in patients diagnosed during infancy or early childhood but recent data indicate that some of these patients remain asymptomatic for long periods [5]. This was true in our cases; the first patient was presented during the adolescence and the second one was diagnosed during childhood. It may also be associated with Caroli’s disease (CD) [2].

The majority of these patients are complicated by cholangitis, biliary sepsis or pancreatitis and until now, all of the CHF patients who have undergone OLT were associated with CD [6, 7].

Our patients had no history of fever and jaundice nor any evidence of cholangitis; both of them, however, developed cirrhosis and hepatic failure needing liver transplantation. Therefore, we could not explain the cause of cirrhosis by repeated cholangitis.

To the best of our knowledge, so far, no case of pure CHF has been reported who needs OLT because of liver failure and clinical signs of cirrhosis. Some of the investigators have described four clinical forms for CHF: portal hypertensive, cholangitic, mixed portal hypertensive-cholangitic, and latent forms [8].

Our cases may add another new category to these four known clinical types of CHF, which is “liver failure and cirrhosis secondary to extensive fibrosis.” However, more cases should be studied before a definite conclusion being made.
